# hiPSC-CM Monolayer Maturation State Determines Drug Responsiveness in High Throughput Pro-Arrhythmia Screen

**DOI:** 10.1038/s41598-017-13590-y

**Published:** 2017-10-23

**Authors:** André Monteiro da Rocha, Katherine Campbell, Sergey Mironov, Jiang Jiang, Lakshmi Mundada, Guadalupe Guerrero-Serna, José Jalife, Todd J. Herron

**Affiliations:** 1 University of Michigan, Internal Medicine-Cardiology, Center for Arrhythmia Research, Ann Arbor, MI 48109 USA; 2 Frankel Cardiovascular Center, Cardiovascular Regeneration Core Laboratory, University of Michigan, Ann Arbor, MI 48109 USA; 3 Centro Nacional de Investigaciones Cardiovasculares Carlos III (CNIC), 28029 Madrid, Spain; 4 CIBER of Cardiovascular Diseases (CIBERCV), ISCIII, Madrid, Spain

## Abstract

Human induced pluripotent stem cell derived cardiomyocytes (hiPSC-CMs) offer a novel *in vitro* platform for pre-clinical cardiotoxicity and pro-arrhythmia screening of drugs in development. To date hiPSC-CMs used for cardiotoxicity testing display an immature, fetal-like cardiomyocyte structural and electrophysiological phenotype which has called into question the applicability of hiPSC-CM findings to the adult heart. The aim of the current work was to determine the effect of cardiomyocyte maturation state on hiPSC-CM drug responsiveness. To this end, here we developed a high content pro-arrhythmia screening platform consisting of either fetal-like or mature hiPSC-CM monolayers. Compounds tested in the screen were selected based on the pro-arrhythmia risk classification (Low risk, Intermediate risk, or High risk) established recently by the FDA and major stakeholders in the Drug Discovery field for the validation of the Comprehensive *In vitro* Pro-Arrhythmia Assay (CiPA). Here we show that maturation state of hiPSC-CMs determines the absolute pro-arrhythmia risk score calculated for these compounds. Thus, the maturation state of hiPSC-CMs should be considered prior to pro-arrhythmia and cardiotoxicity screening in drug discovery programs.

## Introduction

Over the last decade the International Conference on Harmonization’s (ICH) guideline has dictated the type of pre-clinical *in vitro* assays used to determine cardiotoxicity and pro-arrhythmia liability for drug compounds in development. The S7B ICH guidelines put emphasis on screening compounds early in drug development for I_Kr_ current blockade using heterologous cells that overexpress just the hERG (Kv11.1) potassium channel. Although these guidelines have prevented potentially arrhythmogenic drugs from entering the market, the emphasis on the single ion channel (hERG, I_Kr_) assay without consideration of the integrated multi-channel nature of the human cardiac cell and action potential has most likely led to falsely labelling safe and effective drugs as pro-arrhythmic^1,2^. Mislabeling compounds in early development phases of drug discovery has led to cessation of development of potentially safe and effective drugs, and thus the pharmaceutical industry and patients are missing out on opportunities for new therapies^3^. False positive findings for cardiotoxicity and proarrhythmia liability of novel drug candidates may also be due to discordance between a compound’s effects in animals and animal cells compared to those in humans^4^. Ideally pre-clinical cardiotoxicity and pro-arrhythmia screening should be determined using human cardiomyocytes or mechanically and electrically syncytial monolayers of human cardiomyocytes. Alternatively human cardiomyocytes can also be used to form 3-dimensional (3D) tissues^5,6^ and the addition of non-myocyte cells in 3D bioengineered human cardiac tissues has been shown to promote more natural growth and maturation^7^.

Human based pre-clinical cardiotoxicity approaches are currently being developed and validated via collaboration between academic laboratories, federal regulatory agencies and the pharmaceutical industry^4,8–10^. The evolving human based approach for pre-clinical cardiotoxicity is called the Comprehensive *in vitro* Proarrhythmia Assay (CiPA for short). Human induced pluripotent stem cell derived cardiomyocytes (hiPSC-CMs) are central to CiPA as a human model system that may be used in place of the standard hERG assay for pre-clinical cardiotoxicity testing^4,11–13^. hiPSC-CMs can be generated in nearly unlimited quantities, can be cryopreserved and are now offered by several commercial vendors^14–16^. Importantly hiPSC-CMs possess the complex array of ion channels that make up the cardiac action potential, which offers a naturally integrated system with advantages over heterologous cell systems in which a single ion channel is tested^13^. Moreover, human stem cell derived cardiomyocytes can be used to form electrically coupled syncytia of monolayers that can be used to quantify electrical impulse propagation and study mechanisms of re-entrant arrhythmias^17,18^.

**Figure 1:**
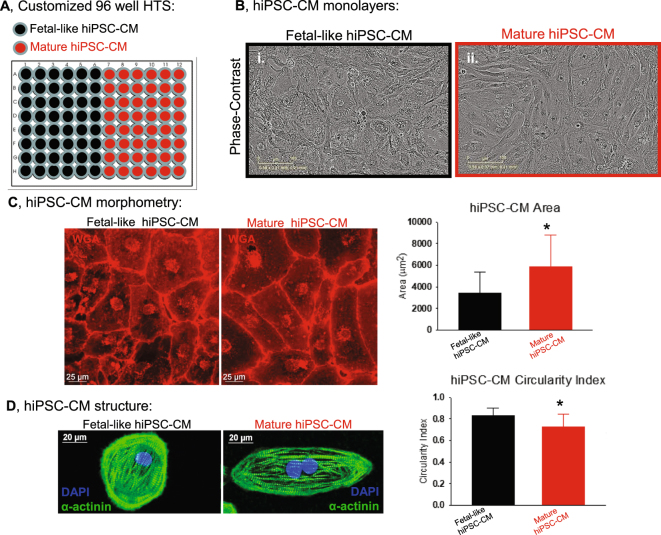
Maturation of hiPSC-CM phenotype in 96-well plates. (**A**) 96-well plates were generated to promote a fetal-like phenotype (black) or mature phenotype (red). (**B**) Phase contrast images of confluent monolayers of (i) fetal-like or (ii) mature phenotype hiPSC-CMs. (**C**) Wheat Germ Agglutinin staining (WGA, red) to determine morphology of fetal-like or mature hiPSC-CMs. Post image acquisition analysis for hiPSC-CM circularity index and hiPSC-CM size (area) was quantified: mature hiPSC-CMs were less circular (circularity index = 0.73 ± 0.12 vs. 0.83 ± 0.07; n = 75) and also were hypertrophied compared to fetal-like hiPSC-CMs (5,841 ± 2984.1 vs. 3,419.1 ± 1950.9 µm^2^; n = 75). Data is mean ± standard deviation, *Denotes significant difference, unpaired t-test; P = 2.6 × 10^−8^. (**D**) Immunostaining and confocal imaging show rod shaped morphology and bi-nucleation of mature hiPSC-CMs. *Denotes significant difference between groups, unpaired t-test, P = 4 × 10^−10^.

However a limitation of the current state of the field involves the maturity of hiPSC-CMs^19–21^. Recent publications have relied on extracellular matrix hardness to mature the phenotype of single hiPSC-CMs and purified monolayers of hiPSC-CMs^20,22^. Yet, the impact that maturation state of hiPSC-CMs has on responsiveness to drug compounds us unclear. Recently we showed that the maturation state of hiPSC-CMs impacts on the ion channel expression profile and electrophysiological phenotype of human cardiac monolayers^22^. Extracellular matrix mediated maturation of hiPSC-CM monolayers significantly induced expression of mature cardiomyocyte markers including cardiac specific troponin I (cTnI), sodium channel (Na_V_1.5), inward rectifier potassium channel (Kir2.1) and connexin43 (Cx43). Furthermore, extracellular matrix mediated maturation induced functionally mature hiPSC-CM monolayers with rapid impulse propagation velocity (up to 50 cm/s), rapid action potential upstroke (up to ~150 V/s) and hyperpolarized resting membrane potential (−78 mV). In the current study we have applied extracellular matrix mediated maturation of hiPSC-CM monolayers plated in 96-well plates for high throughput assessment of drug effects on action potential duration using spontaneously beating and electrically paced preparations. Here we generated customized 96-well plates in which half of the monolayers were fetal-like and the other half were mature hiPSC-CMs (Fig. 1). Using a subset of CiPA validated compounds^4^ classified as low risk (diltiazem and tamoxifen), intermediate risk (cisapride, clozapine, terfenadine) or high risk (bepridil, dofetilide, E-4031, quinidine) for causing Torsade de Pointes (TdP) we determined the effect of maturation state on hiPSC-CM monolayer responsiveness. Results indicate that the maturation state of hiPSC-CM monolayers influences the electrophysiological responsiveness and thus should be considered when using hiPSC-CMs as part of CiPA for drug discovery projects.

## Methods

### Maturation of Commercially Available hiPSC-CM Monolayers

hiPSC-CMs were used from two different commercial sources: Cellular Dynamics International (CDI, Madison WI) and Cellartis (Takara Bio USA). Custom made 96-well plates with inserts of PDMS (Polydimethylsiloxane**)** were generated essentially as before^22^. PDMS vulcanized silicone transparent sheeting was obtained from SMI (Specialty Manufacturing, Inc, Saginaw, MI) with 40D (D, Durometer or ≈1000 kPa) hardness and 9 mm diameter coverslips were cut out to fit in each well of 96 well plates. PDMS coverslips were manually added to each well of 96 well plates (Corning) and each plate was subsequently sterilized via ethylene oxide sterilization. PDMS coverslips were then matrigel-coated (500 μg/mL; BD Biosciences, San Jose, CA) prior to plating hiPSC-CMs. Thawing and plating of cryopreserved vials of cells into 96-well plates was as essentially described before^22^. Briefly, 5 × 10^4^ cells were plated per well and allowed to form continuous monolayers and maintained in RPMI (Life Technologies) media supplemented with B27 + insulin (Life Technologies). Monolayers were maintained in culture for seven days before electrophysiological testing. Immature fetal-like hiPSC-CM monolayers were generated in 96 well plates without the PDMS coverslip inserts. Fetal-like hiPSC-CMs were also plated on matrigel coated plastic bottoms.

**Figure 2:**
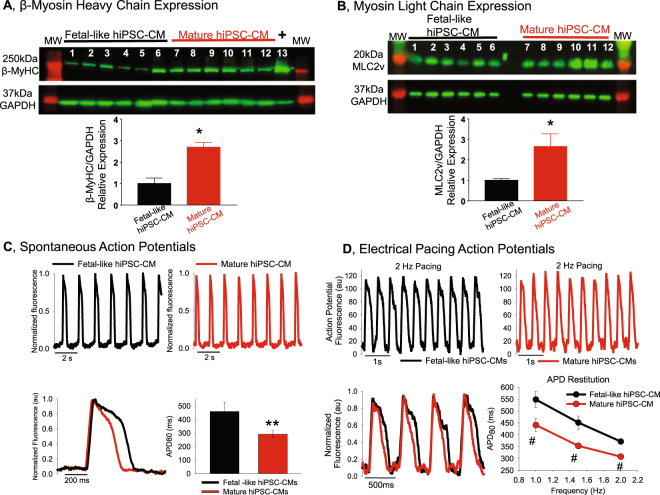
Phenotype analysis of fetal-like and mature hiPSC-CMs. (**A**) Western blotting for the mature ventricular myocyte marker β-MyHC. Lanes 1–6 are independent monolayers of fetal-like hiPSC-CMs, lanes 7–12 are independent monolayers of mature hiPSC-CMs. Lane 13 is a positive control human ventricular heart homogenate. This represents one blot where each group was loaded on the same gel and antibody incubation performed at the same time. GAPDH signal was used to normalize for any uneven protein loading. MW = molecular weight marker, *Indicates significant difference, P = 0.0014, unpaired t-test. (**B**) Western blotting for ventricular myosin light chain (MLC2v), loading sequence is similar to panel A, *Indicates significant difference, P = 0.013, unpaired t-test. (**C**) Spontaneous action potentials recorded using optical mapping (FluoVolt, transmembrane voltage dye) reveal that mature hiPSC-CM monolayers have abbreviated action potential duration 80 (APD_80_, ms; mature = 291.8 ± 8.8 ms; n = 8 vs. fetal-like = 459.5 ± 24.3 ms; n = 8). *P = 0.000014 unpaired t-test. (**D**) Electrical pacing also reveals abbreviated action potential duration in the mature hiPSC-CM monolayers. Original recordings of electrically paced (2 Hz) monolayers of fetal-like (black) and mature (red) hiPSC-CMs. APD restitution plots show the quantification at three different frequencies of activation (1 Hz, 1.5 Hz and 2.0 Hz). Mature hiPSC-CMs had abbreviated APD_80_ at each frequency tested (1.0 Hz:fetal-like APD_80_ = 548.7 ± 33.8 ms; n = 7 vs. mature APD_80_ = 441.2 ± 13.3 ms; n = 5) (1.5 Hz:fetal-like APD_80_ = 451.5 ± 27.5 ms; n = 7 vs. mature APD_80_ = 353.1 ± 12.8 ms; n = 5) (2.0 Hz: fetal-like APD_80_ = 371.8 ± 11.3 ms; n = 7 vs. mature APD_80_ = 308.0 ± 11.3 ms; n = 5). Data expressed as mean ± sem; unpaired t-test significance ^#^P ≤ 0.02.

Confluent monolayer formation was monitored and confirmed using time-lapse microscopy (Incucyte Zoom, Essen Bioscience, Ann Arbor, MI). Figure 1 shows an example of confluent monolayers using CDI cardiomyocytes. hiPSC-CM morphology was quantified using Wheat Germ Agglutinin conjugated to AlexaFluor 594 (WGA, Thermo Fisher Scientific) with subsequent confocal imaging. In each condition (fetal-like and mature hiPSC-CM monolayers) WGA staining was used to quantify the cell area and perimeter and subsequently the circularity of iPSC-CMs within the monolayers was calculated using the equation: Area*π*4/Perimeter^2^. By this equation the circularity of a circle is 1. Further characterization of molecular maturation was performed by Western blotting for ventricular β-myosin heavy chain (β-MyHC, ATCC A4.951 mouse monoclonal antibody) and ventricular myosin light chain 2 (MLC2v, Protein Tech Group) shown in Fig. 2. These antibodies have been used extensively for quantification of myofilament protein expression. For reliable comparison and quantification each group was loaded on the same gel, and probed with the same antibodies on the same membrane. These complete blots which include molecular weight markers and a positive control (Fig. 2A, human cardiac ventricular homogenate, kind gift from Dr. Adam Helms). For each protein the membrane was sectioned according to molecular weight prior to antibody incubation to enable probing for multiple proteins on the same membrane.

### Purification and Maturation of hiPSC-CMs Differentiated Using the Small Molecule Approach

In parallel experiments purified hiPSC-CM monolayers were generated in our laboratory using the established BJ-iPSC line, which was derived using mRNA reprogramming^22,23^. Pluripotent stem cell experiments were performed with approval of the HPSCRO (Human Pluripotent Stem Cell Research Oversight Committee) of the University of Michigan. iPSCs were maintained in feeder free conditions (on matrigel) in xeno-free iPS Brew Media (Miltenyi Biotec, Germany) with manual marking and picking to remove spontaneous differentiation. Cardiac directed differentiation was performed as previously described using the small molecule based approach now commonly referred to as the GiWi protocol^14,22,24^. Briefly, pure hiPSCs were singularized using versene, plated as monolayers in 6 well plates (1 × 10^6^ hiPSCs/well) and maintained in stem cell media until reaching ~95% confluence. On day 0 of this protocol cardiac directed differentiation was initiated by culturing stem cell monolayers in RPMI media supplemented with B27 (-insulin) and the glycogen synthase kinase-3 inhibitor CHIR99021 (12 µM). On day 1 CHIR99021 was removed and cells are cultured in RPMI with B27 (-insulin) until day 3. On day 3 the small molecule Wnt inhibitor IWP4 (5 µM) was added to the cells and not removed until day 5. Days 5–7 the cells are maintained in RPMI with B27 (-insulin). On day 7 the RPMI media was supplemented with B27+ insulin and maintained in this media with daily changes until day 30. On day 30 of directed differentiation cell cultures were trypsinized and cardiomyocytes were purified using magnetic activated cell sorting to target and deplete non-myocytes from the cell population which resulted in cardiomyocytes enriched to ~98% purity (Miltenyi Biotec, PSC-derived cardiomyocyte isolation kit, human). Purified hiPSC-CMs were plated in 96-well plates for maturation as described above for commercially available cell lines.

### High Content 96-Well Plate Optical Mapping for hiPSC-CM Action Potentials

Optical mapping was performed as essentially described recently^22^ with slight modification. A schematic of the 96-well plate optical mapping set up is shown in supplemental Fig. 1. In this assay a 96-well plate is positioned below a high speed CCD camera (200 fps, 80 × 80 pixels) on a heating block to enable recording at physiological temperature (37 °C). hiPSC-CM monolayers were loaded with the membrane potential dye called FluoVolt™ which is commercially available (ThermoFisher Scientific, F10488). Blue LED illumination was used and spontaneous or electrically excited action potentials were recorded with the CCD camera via appropriate emission filter (515 nm, green light, Chroma). For calcium flux quantification monolayers were loaded with fluo-4AM (10 µM). In this assay a 6 × 6 array of wells (supplemental Fig. 1C, 36 wells total) in the 96-well plate could be imaged simultaneously to enable rapid dose response data for all compounds using up to six different doses. Post-acquisition data analysis was done using custom software (Scroll)^22,25^ with automated peak detection capability and automated repolarization quantification to determine APD_80_ (action potential at 80% of repolarization). Supplemental Fig. 2 shows typical action potential recordings and automated peak and repolarization detection. In some experiments electrical pacing was done to control for rate of activations (supplemental Fig. 3). A custom made frame of field stimulation electrodes was engineered to enable electrical pacing to the 6 × 6 array of wells. Supplemental Fig. 4 shows the successful use of electrical pacing to match the rate of activation in multiple wells.

### Compound formulation and application to hiPSC-CM monolayers

Compounds were obtained from the SCREEN-WELL® Cardiotoxicity library (Enzo LifeScience Inc., BML-2850–0100) or through Sigma. Compounds obtained from the SCREEN-WELL® library (low risk = diltiazem, tamoxifen; intermediate risk = terfenadine, clozapine, cisapride; high risk = bepridil, quinidine, dofetilide) were diluted in DMSO as 10 mM stock and serially diluted to the indicated concentrations in HBSS (Hank’s Balanced Salt Solution, Gibco #14025-092). Compounds not on the CiPA list, including amiodarone, nilotinib and doxorubicin also were part of the SCREEN-WELL® Cardiotoxicity library and were handled in the same way. Fluoxetine was obtained from Sigma (fluoxetine HCl-F132) and was diluted in DMSO to make 10 mM stock, then further diluted in HBSS at the indicated concentrations for screening experiments. On the day of experimentation maintenance media was removed from the monolayers, which then were loaded with FluoVolt (per manufacturer protocol in HBSS) and subsequently treated with test compounds diluted in HBSS for 30 min prior to action potential data acquisition. Action potentials were recorded in the presence of each test compound.

## Results and Discussion

### hiPSC-CM Monolayer Maturation

Data in Figs 1 and 2 demonstrate maturation of the structural and functional phenotype of hiPSC-CMs following seven days in primary culture. Consistent with previous work^22^ mature hiPSC-CMs were larger in cross-sectional area (Fig. 1C), indicative of cellular hypertrophy which occurs as fetal-like cardiomyocytes mature and growth switches from hyperplastic to hypertrophic^26^. In addition, mature hiPSC-CMs were less circular (more rod-shaped) and also had evidence of bi-nucleation compared to fetal-like hiPSC-CMs (Fig. 1D), as described recently^22^. Although bi-nucleation was more apparent in mature monolayers, the frequency of bi-nucleation quantified by flow cytometry is relatively low (~35.24 ± 3.0% in mature monolayers vs 21.68 ± 1.08% in fetal-like monolayers)^22^. Myofilament markers of mature ventricular cardiomyocytes (β-MyHC and MLC2v) were elevated in mature hiPSC-CM monolayers (Fig. 2A and B). Functionally mature monolayers had abbreviated action potential duration in spontaneously beating (Fig. 2C) as well as electrically paced CDI hiPSC-CM monolayers (Fig. 2D). The action potential profile of hiPSC-CM monolayers from Cellartis cells could also be abbreviated using the same approach (supplemental Fig. 5).

### Maturation state impact on responsiveness to hERG channel blockers

#### E-4031

**Figure 3:**
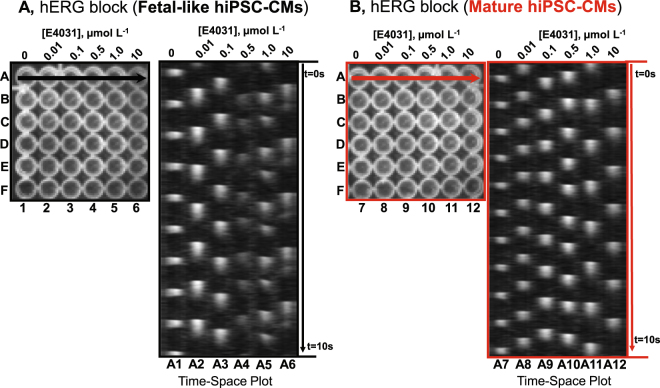
Multi-well optical mapping and E-4031 Dose Response. (**A**) In each experiment presented a 6 × 6 array of wells of a 96-well plate were recorded from simultaneously. Here six doses of the specific hERG channel blocker, E-4031 was added to each well of a single column at the specified concentrations. For example, column 1 = 0, vehicle control, column 2 = 0.01 µmol L^−1^ and each column has elevating concentrations up to column 6 = 10.0 µmol L^−1^. The time space plot is made from drawing a line across row A (indicated by black horizontal arrow) and the spontaneous activations at each dose are shown. Here time 0 (t = 0 s) is at the top and the entire movie recording was 10 s in duration. E-4031 progressively increased APD and the occurrence of EADs are apparent. (**B**) The same experimental set up as in panel A except here mature hiPSC-CM monolayers were used (red). In mature monolayers APD prolongation could be observed, but with lower magnitude of prolongation and without the occurrence of EADs.

**Figure 4:**
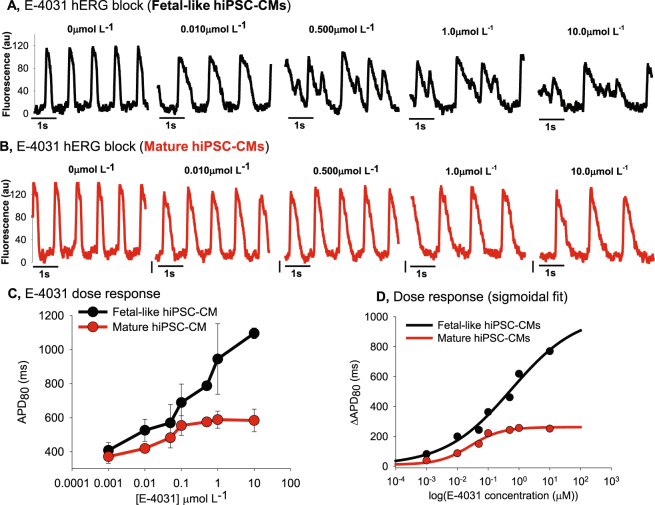
Quantification of E-4031 effect on hiPSC-CM fetal-like or mature monolayers. (**A**) Single pixel recordings of spontaneous action potentials in fetal-like hiPSC-CM monolayers with treatment of indicated concentrations of E-4031. (**B**) Single pixel recordings of spontaneous action potential in mature hiPSC-CMs with treatment of indicated concentrations of E-4031. (**C**) Dose response plot showing that maturation state of hiPSC-CMs impacts on spontaneous action potential responsiveness to E-4031. (**D**) E**-**4031 dose response data for each condition was fit using the Sigmoidal, Hill, 4 Parameter equation using Sigmaplot software: f = y0 + a*x^b^/(c^b^ + x^b^); R^2^ = 0.98. Here the drug induced change of absolute APD_80_ (ΔAPD_80_) is plotted as a function of dose.

It has been reported that immature hiPSC-CMs rely critically on the hERG channel for action potential repolarization and setting the maximum diastolic potential^27^. We recently showed that mature hiPSC-CMs also express other repolarizing potassium currents such as I_K1_ and that calcium transients of mature hiPSC-CMs are less sensitive to hERG blockade with E-4031. Here we have constructed more complete dose response curves to determine the effect of E-4031 to prolong the action potential of fetal-like or mature hiPSC-CM monolayers. Figure 3 shows original recordings in the form of time-space plots (similar to a confocal line scan) of action potentials over several doses of E-4031 in fetal-like (black, panel A) and mature hiPSC-CM monolayers (red, panel B). These time-space plots reveal the prolongation of the action potential duration and also induction of early after depolarizations (EADs; shown as double depolarizations at the largest drug doses) in the fetal-like hiPSC-CM monolayers (panel A). On the other hand, in mature hiPSC-CM monolayers (red, panel B) APD prolongation could be detected at low concentrations but there was much less evidence of EADs. In Fig. 4A, single pixel recordings over time show the differential response to E-4031 based on maturation state of CDI hiPSC-CM monolayers, which was also observed in the BJ-hiPSC-CM purified monolayers generated in our laboratory (supplemental Fig. 6), although using the BJ-hiPSC-CMs EADs could be induced at the highest dose of E-4031. Next, to match pacing frequencies we utilized the custom made electrical pacing frame for 96-well plates to further test the function of mature iCell hiPSC-CM monolayers. Supplemental Fig. 7 shows a similar qualitative result during electrical pacing (1 Hz) where EADs were very apparent in the fetal-like cardiac monolayers while EADs were not apparent in the mature monolayers. The dose response effect of E-4031 to prolong the action potential duration is summarized in Fig. 4C and D showing that maturation state largely determines the absolute monolayer response during spontaneous recordings, as well as with electrical pacing (1 Hz, supplemental Fig. 7). Further confirmation experiments in mature hiPSC-CM monolayers was done using a compound with well-known high risk for causing APD prolongation (quinidine, supplemental Fig. 8) and a compound well-known for low risk for causing APD prolongation (tamoxifen, supplemental Fig. 9). The compounds had the expected effect on APD, thus confirming that the mature monolayer platform will likely predict risk of a compound for APD prolongation.

#### Dofetilide

**Figure 5:**
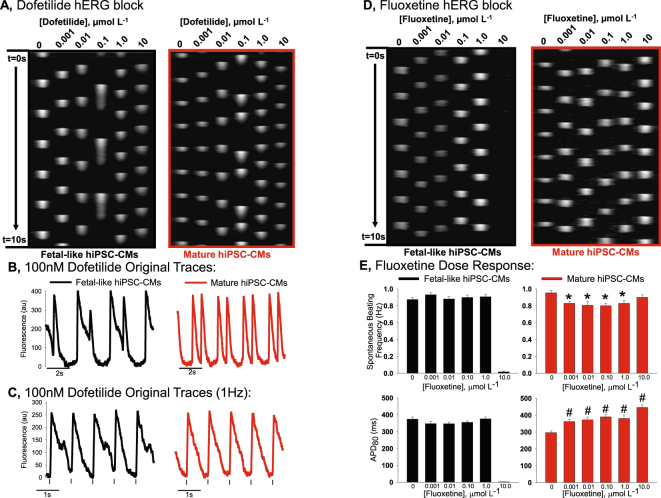
Effect of maturation state on hERG blockade using dofetilide and fluoxetine. (**A**) Time-space plots for dofetilide effect on spontaneously beating fetal-like (black) or mature hiPSC-CMs (red) monolayers. Dose of dofetilide ranged from 0 to 10 µmol L^−1^. (**B**) Single pixel recordings of hiPSC-CM monolayers treated with 100 nmol L^−1^ dofetilide shows greater effect to prolong the action potential of fetal-like hiPSC-CM monolayers. (**C**) Single pixel recordings of electrically paced hiPSC-CM monolayers show APD alternans in fetal-like monolayers (black), but not in mature monolayers (red). (**D**) Time space plot showing effect of fluoxetine in fetal-like vs. mature hiPSC-CMs. (**E**) Effect of fluoxetine on spontaneous beating rate and APD_80_ of fetal-like or mature hiPSC-CM monolayers. *Indicates significantly different frequency from vehicle control, unpaired t-test P < 0.002. ^#^Indicates significantly different APD_80_ from vehicle control, unpaired t-test P < 0.0002.

Next we determine the effect of hiPSC-CM monolayer maturation state on responsiveness to dofetilide, another specific blocker of the hERG channel. Results are similar to those observed for E-4031 treatment. Figure 5A shows original recordings of spontaneous action potentials of fetal-like or mature hiPSC-CM monolayers over several doses of dofetilide. Similar to the E-4031, the mature hiPSC-CM monolayers responded differently than the fetal-like hiPSC-CM monolayers. From Fig. 5A and B it is apparent that the mature hiPSC-CM monolayers did not have as severe action potential duration prolongation as observed in the fetal-like monolayers. Furthermore, as shown in Fig. 5C, with electrical pacing (1 Hz) action potential duration alternans were apparent in fetal-like monolayers, but not in the mature hiPSC-CM monolayers when each was incubated in the same concentration of dofetilide (100 nM).

#### Fluoxetine

Fluoxetine is a compound with hERG channel blocking effects^28^ as well as other off target ion channel block, including: Nav1.5^29^, and voltage gated calcium channels^30^. We found differential effects of fluoxetine when comparing fetal-like to mature hiPSC-CM monolayers. This is summarized in Fig. 5D and E. Specifically, in fetal-like monolayers we did not detect any APD_80_ prolongation, nor alteration of the spontaneous beating frequency at sub-maximal doses (below 10.0 µmol L^−1^). However, 10.0 µmol L^−1^ fluoxetine caused quiescence in 100% of fetal-like monolayers tested (8/8). This finding is consistent with a recent report using immature hiPSC-CM monolayers which showed no effect of fluoxetine to prolong the action potential duration, but quiescence at high dose. On the other hand, fluoxetine prolonged the action potential duration in mature monolayers even at the lowest concentration (1.0 nmol L^−1^), slowed the spontaneous beating rate and did not induce quiescence at any concentration tested. Thus, unlike other compounds tested mature hiPSC-CM monolayers are more sensitive to the effects of fluoxetine to prolong the action potential duration than immature monolayers, although fetal-like monolayers’ electrophysiological function was completely inhibited at the highest concentration.

#### Terfenadine

Terfenadine is an antihistamine compound that also blocks the hERG channel Terfenadine is classified as a compound with intermediate risk to cause QT prolongation of the human ECG. Supplemental Figure 10 shows that fetal-like hiPSC-CM monolayers are more sensitive to terfenadine’s effect of prolonging the action potential duration.

### Other compounds

#### Nilotinib

**Figure 6:**
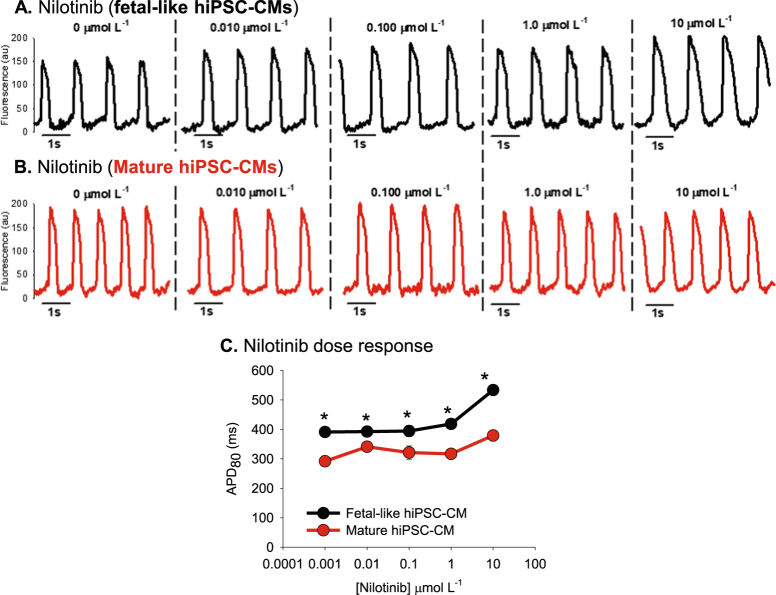
Nilotinib effects on hiPSC-CM monolayer electrophysiological function. (**A**) Original recordings using fetal-like hiPSC-CM monolayers. (**B**) Original recordings using mature hiPSC-CM monolayers. (**C**) Dose response showing differential effect of nilotinib on fetal-like and mature hiPSC-CM monolayers action potential duration. *Denotes statistically significant difference between fetal-like and mature hiPSC-CM APD_80_ values; unpaired t-test; n = 4 per dose; P < 0.04.

**Figure 7:**
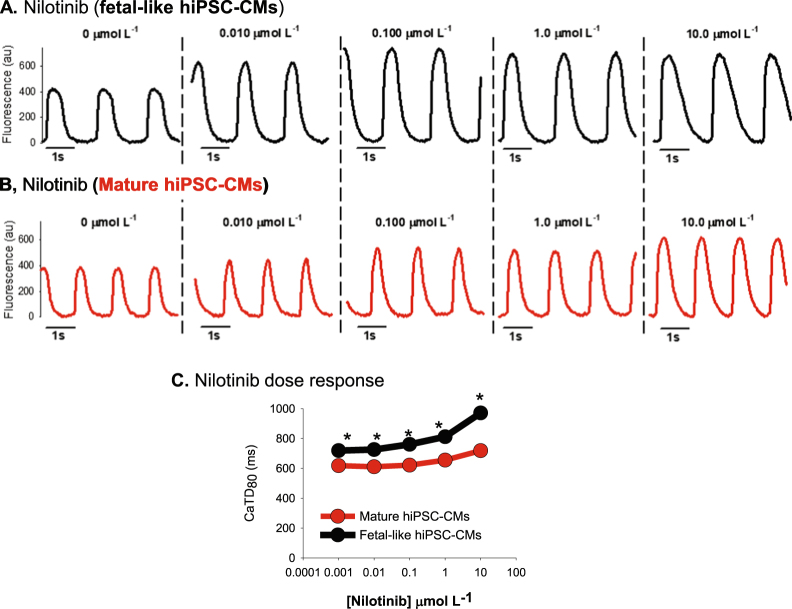
Nilotinib effects on hiPSC-CM intracellular calcium flux. (**A**) Original recordings of intracellular calcium flux in fetal-like (black) and mature hiPSC-CM monolayers (**B**, red traces). (**C**) Nilotinib increased calcium transient duration 80 (CaTD_80_) in a dose dependent manner. Similar to the APD recorded in Fig. 6, CaTD_80_ is prolonged in fetal-like compared to mature hiPSC-CM monolayers at each dose of nilotinib tested. *Denotes statistically significant difference between fetal-like and mature hiPSC-CM CaTD_80_ values; unpaired t-test; n = 4 per dose; P < 0.05.

Nilotinib is an anti-cancer kinase inhibitor compound with possible side effects to prolong the QT interval. We tested the effect of nilotinib on hiPSC-CMs and the results are presented in Figs 6 and 7. Figure 6 shows action potential recordings from fetal-like (black traces) and mature hiPSC-CM monolayers (red). In both maturation states APD prolongation was detected, although to a greater extent in the fetal-like monolayers. In Fig. 7 calcium transients were recorded using fluo-4AM loaded monolayers to study the effect of nilotinib on intracellular calcium flux dynamics. Calcium transient duration (CaTD80) was prolonged in the fetal-like hiPSC-CM monolayers compared to the mature monolayers (727.7 ± 20.5 ms vs. 532.4 ± 20.9 ms; n = 4 monolayers per group) at baseline and this difference between groups persisted over the doses of nilotinib tested. The abbreviated calcium transient duration in the mature hiPSC-CM monolayers suggests also a greater degree of maturation of the calcium homeostasis mechanisms that regulate cardiomyocyte calcium flux. These data show the utility of this platform to determine compound effects on intracellular calcium flux as well as action potential duration.

#### Amiodarone

Amiodarone is an anti-arrhythmic compound used to treat arrhythmias like ventricular tachycardia and atrial fibrillation due to its effect to prolong the action potential duration^31^. It is a compound with complex multi-channel blocking effects including inward as well as outward currents^32^. Shown in Table 1, amiodarone surprisingly shortened action potential duration in fetal-like cardiomyocytes. On the other hand, amiodarone had the anticipated effect to prolong the APD in mature hiPSC-CM monolayers. The maturation dependent effects for amiodarone suggest that ion channels necessary to cause APD prolongation may not be present in the fetal-like hiPSC-CM monolayers.

**Table 1:** APD_80_ Prolongation Risk Scores.

*Compound*	*Maximum APD*_*80*_ *Prolongation Risk Score (Fetal-like hiPSC-CM)*	*Maximum APD*_*80*_ *Prolongation Risk Score (Mature hiPSC-CM)*
***CiPA: High Risk***		
***Bepridil:***	**—**	**1.64**
***Dofetilide:***	**2.14**	**1.81**
***Quinidine:***	**1.91**	**1.81**
***E-4031:***	**3.04**	**2.04**
***Mean:***	**2.36 ± 0.3**	**1.83 ± 0.08**
***Range:***	**1.91–3.04**	**1.64–2.04**
***CiPA: Intermediate Risk***		
***Cisapride:***	**1.49**	**1.15**
***Clozapine:***	**1.14**	**1.33**
***Terfenadine:***	**1.43**	**1.21**
***Mean:***	**1.35 ± 0.10**	**1.23 ± 0.05**
***Range:***	**1.14–1.49**	**1.15–1.33**
***CiPA: Low Risk***		
***Diltiazem:***	**0.97**	**1.03**
***Tamoxifen:***	**1.12**	**1.04**
***Mean:***	**1.05 ± 0.08**	**1.04 ± 0.04**
***Range:***	**0.97–1.12**	**1.03–1.04**
***Other Compounds***		
***Oncology***		
***Nilotinib:***	**1.36**	**1.29**
***Doxorubicin:***	**1.15**	**1.15**
***Anti-arrhythmic***		
***Amiodarone:***	**0.91**	**1.34**
***Anti-depressant***		
***Fluoxetine:***	**1**	**1.28**

Values of the maximal effect on APD for each compound, independent of dose. Presented are values calculated for fetal-like monolayers (***italic*** font) or mature monolayers (***bold and underline*** font). In the majority of CiPA classified compounds the values were higher for fetal-like monolayers compared to mature monolayers.

### Compound Risk Stratification

**Figure 8:**
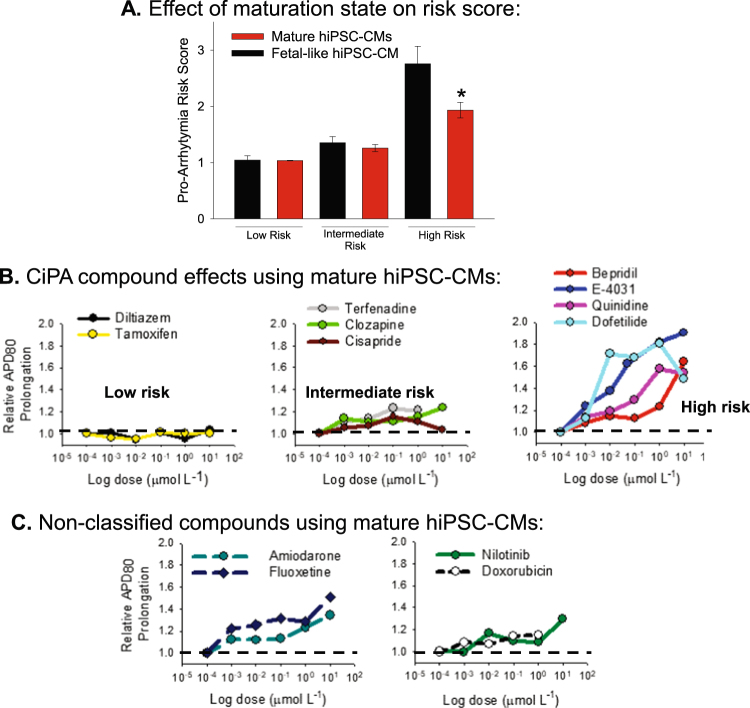
APD Prolongation risk scores. (**A**) Pooled values for the maximal prolongation risk score at any concentration of compound tested in either fetal-like (black) or mature hiPSC-CM monolayers. High risk compounds showed the greatest dependence on maturation state; *P < 0.05, unpaired t-test. Values for each compound are presented also in Table 1. (**B**) CiPA compound APD prolongation risk stratification using mature hiPSC-CM monolayers. For each compound the dose dependence on relative prolongation of APD_80_ is presented. Relative prolongation is calculated by dividing the absolute APD_80_ (ms) by the vehicle control APD_80_ quantified for each 96 well plate. Risk score of 1 = no risk; risk score of >1 = risk of APD prolongation and by inference QT interval. **C**, Non-CiPA compounds APD prolongation risk scores calculated using mature hiPSC-CM monolayers. The dashed line in each panel highlights risk score of 1, which indicates no risk for causing APD prolongation and by inference prolongation of the QT interval and pro-arrhythmia.

The effect of each compound to prolong or shorten the APD_80_ of hiPSC-CM monolayers was quantified relative to vehicle control values obtained in each plate. In other words, the APD_80_ or CaTD_80_ quantified at each concentration of each compound was divided by the APD_80_ or CaTD_80_ quantified for vehicle control wells. Risk score values for each compound are presented in Table 1. Risk score values were quantified for both fetal-like and mature hiPSC-CM monolayers. In most compounds tested the risk score value depended on the maturation state of the hiPSC-CM monolayers. Figure 8A shows the average risk score grouped by CiPA risk category and maturation state. On average the risk score for intermediate and high risk compounds was greater in the fetal-like than in the mature hiPSC-CM monolayers. The dose dependent risk profile for each compound tested using mature hiPSC-CM monolayers is shown in Fig. 8B and provides evidence that risk stratification proposed by the CiPA initiative can be accurately predicted using this HTS platform. Dose dependent risk profiles for unclassified compounds determined using mature hiPSC-CM monolayers are presented in Fig. 8C.

In summary the results of this study show the impact of maturation state of hiPSC-CMs on drug compound responsiveness. Fetal-like hiPSC-CMs appear to be more sensitive to compounds considered to be of intermediate or high risk for causing pro-arrhythmia and APD prolongation. The maturation state of hiPSC-CMs is important to consider when using these cells for pre-clinical pro-arrhythmia and cardiotoxicity screening in drug discovery programs. Furthermore, we have presented a new method for dictating the functional and structural maturation state of hiPSC-CM monolayers. Using this approach, it is possible to test a compound’s effects on either fetal-like or mature human cardiomyocytes and this may add value to pre-clinical toxicity screening by providing a comprehensive examination of drug effects on varying developmental cardiac phenotypes (fetal-like vs. mature adult phenotypes).

### Study Limitations

E-4031 is the only compound tested in which a full concentration curve was generated and fit to a sigmoidal curve, this was the only compound that yielded a maximal effect at the concentrations tested. The other compounds tested at concentrations up to 10 µM did not reach a maximal effect in all cases; this maximal concentration is well above the EC50 concentration for all compounds used in pre-clinical pro-arrhythmia testing. Nevertheless, these concentrations were sufficient to determine effectiveness of the platform to detect action potential prolongation, a surrogate for pro-arrhythmia. Also, comparison with human adult cardiomyocytes obtained from fully formed human hearts would be an ideal comparator for maturation; however adult human cardiomyocytes are not readily available for high throughput screens and are not amenable to formation of electrically coupled monolayers for determination of drug induced pro-arrhythmia. Furthermore, human adult cardiomyocytes are not readily available for study and the viability of these cardiomyocytes isolated typically from transplant or cadaveric hearts is notoriously low.

## Electronic supplementary material

Supplementary Information
