# Supplementary material for: hiPSC-CM Monolayer Maturation State Determines Drug Responsiveness in High Throughput Pro-Arrhythmia Screen
Source: Sci Rep. 2017 Oct 23;7:13834. doi: 10.1038/s41598-017-13590-y (PMC5653750; doi:10.1038/s41598-017-13590-y)
Supplement: Supplementary file 1 — Supplementary Information [file 41598_2017_13590_MOESM1_ESM.pdf]

JOURNAL: *Scientific Reports-Nature Publishing Group*

TITLE: hiPSC-CM Monolayer Maturation State Determines Drug Responsiveness in High Throughput Pro-Arrhythmia Screen

RUNNING TITLE: Mature hiPSC-CMs for pre-clinical pro-arrhythmia screening

AUTHOR INFORMATION

André Monteiro da Rocha*§, Katherine Campbell*, Sergey Mironov*, Jiang Jiang*, Lakshmi Mundada*, Guadalupe Guerrero-Serna*, José Jalife*† and Todd J. Herron*§‡

*University of Michigan, Internal Medicine-Cardiovascular Medicine, Center for Arrhythmia Research, Ann Arbor, MI 48109

§Frankel Cardiovascular Center, Cardiovascular Regeneration Core Laboratory, University of Michigan, Ann Arbor, MI 48109

†Centro Nacional de Investigaciones Cardiovasculares Carlos III (CNIC), 28029 Madrid, Spain; CIBER of Cardiovascular Diseases.

‡Corresponding Author Address: UM-North Campus Research Complex, 2800 Plymouth Road, Bldg 26-223N, Ann Arbor, MI 48109-2800, toddherr@umich.edu, (734)998-0460 (phone), (734)998-7500 (Fax)

Supplemental figures and legends

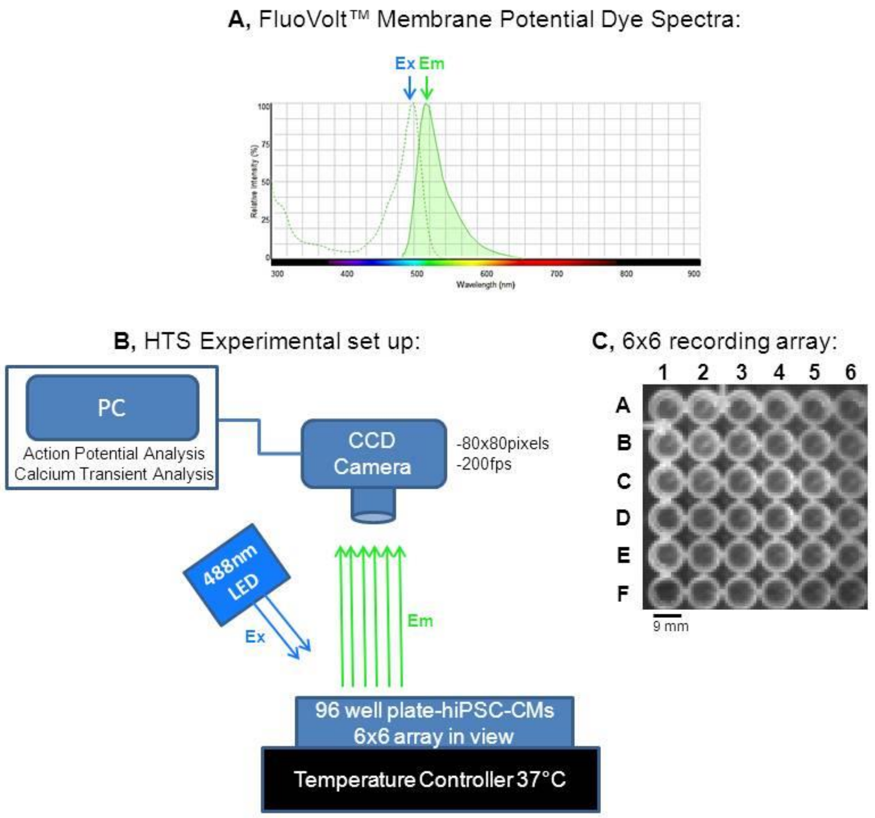

Supplemental figure 1. Configuration for 96 well plate pro-arrhythmia HTS. This platform was used for experiments to compare fetal hiPSC-CM monolayers to mature hiPSC-CM monolayers. Each plate was manually translated into the field of view to enable interrogation of the entire 96 well plate.

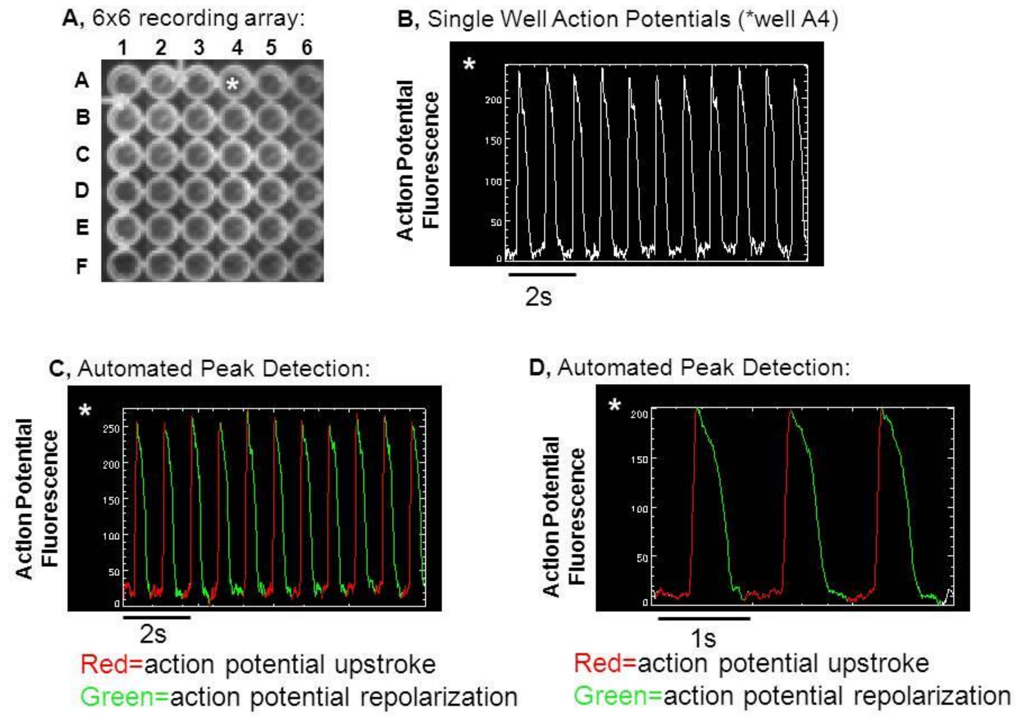

Supplemental figure 2. Examples of data acquired and analysis of action potential recordings. **A,** 6x6 array visualized. **B,** 10s recording of spontaneous action potentials from well A4. **C&D**, Automated peak detection using custom software.

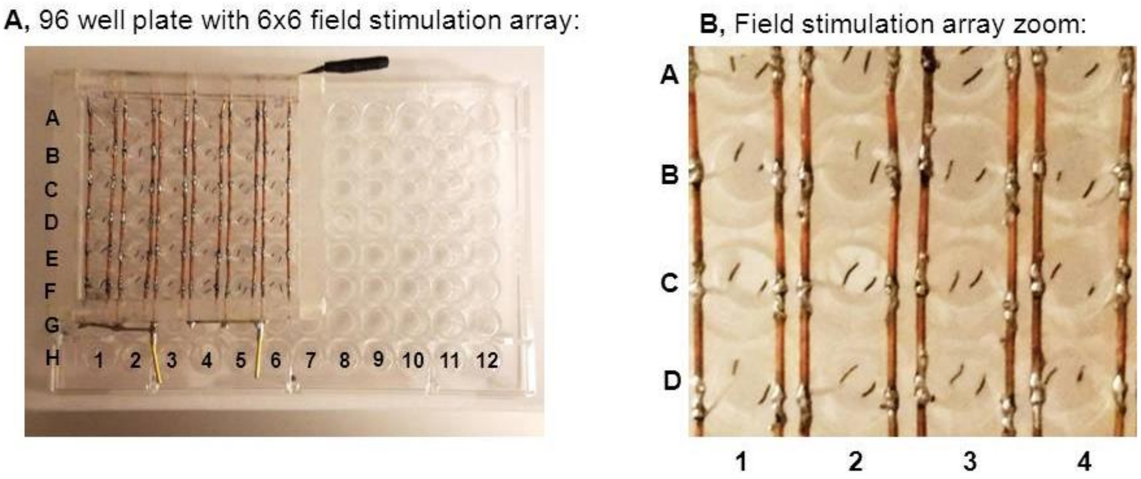

Supplemental figure 3. Custom made field stimulation frame for electrical pacing of 6x6 array of hiPSC-CM monolayers.

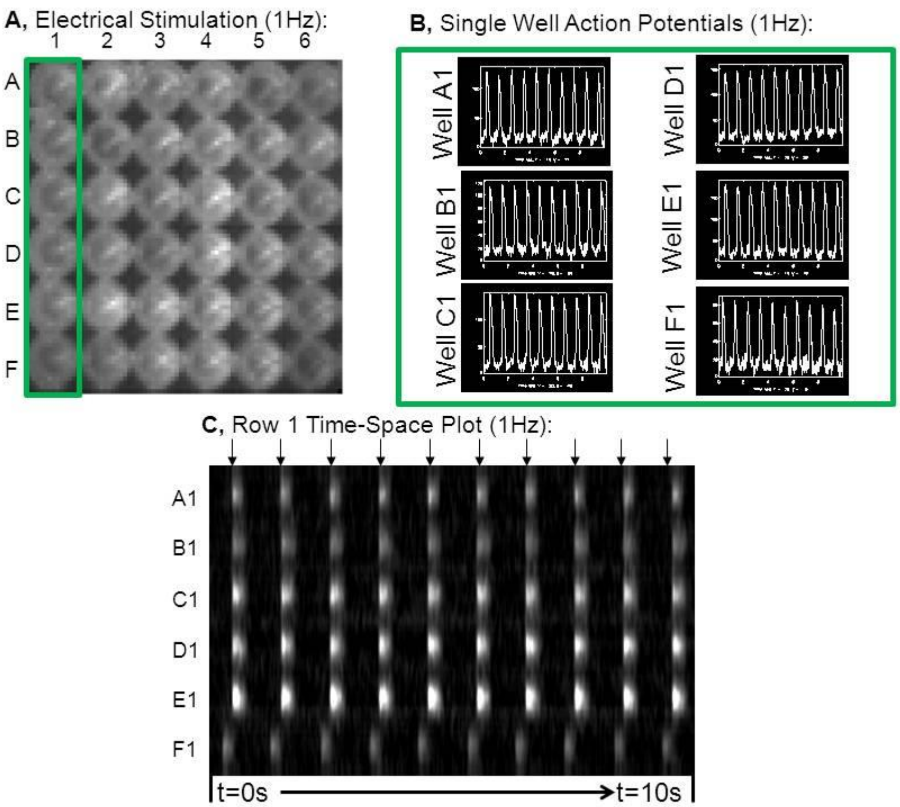

Supplemental figure 4. Example traces of synchronized (1Hz) hiPSC-CM monolayers using electrical pacing frame shown in supplemental figure 3. **A&B,** Representative traces collected for column 1 of this 6x6 array. Single pixel recordings of 10s movie (1Hz pacing) from each well of column 1 show synchronization of activation. **C,** Time space plots for column 1 also show synchronization of activations. arrows indicate electrical stimulation.

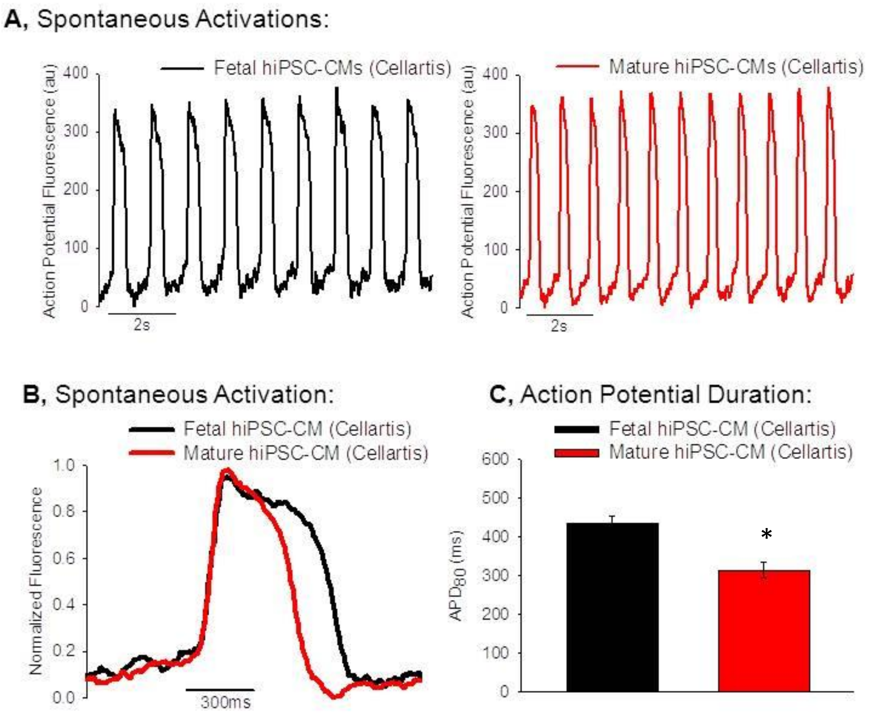

Supplemental figure 5. Maturation of Cellartis commercially available hiPSC-CMs. These are unpurified hiPSC-CM monolayers. **A,** Spontaneous activations of fetal and mature monolayers. **B,** Overlay of spontaneous action potentials shows difference of action potential duration. **C,** APD_{80} is significantly abbreviated in mature hiPSC-CM monolayers;*$P<0.01$, unpaired t-test.

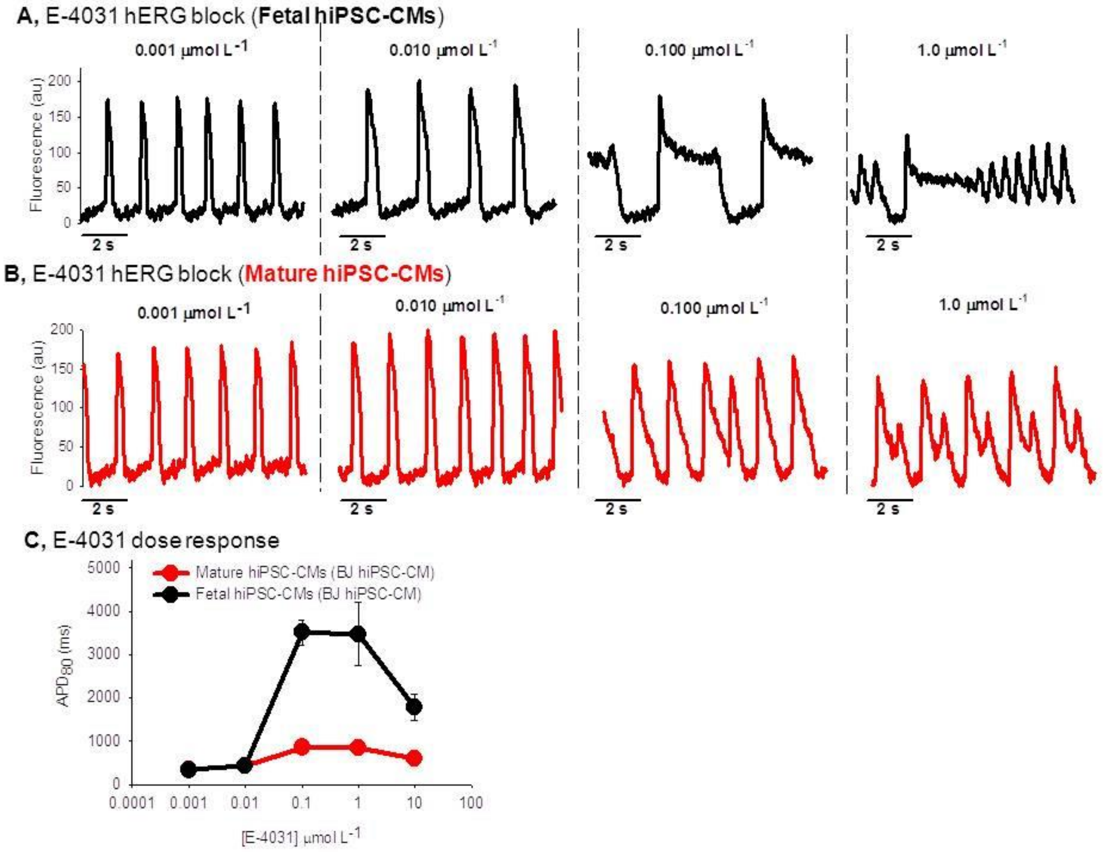

Supplemental figure 6. E-4031 effect in BJ hiPSC-CM monolayers generated using the small molecule approach with magnetic activated cell sorting (MACS) based cardiomyocyte purification. **A&B,** original recordings of action potentials over various doses in fetal (A, black) or mature hiPSC-CM monolayers (B, red). **C,** dose response shows divergent effects of E-4031 in fetal (black) compared to mature monolayers (red).

A, Time space plots

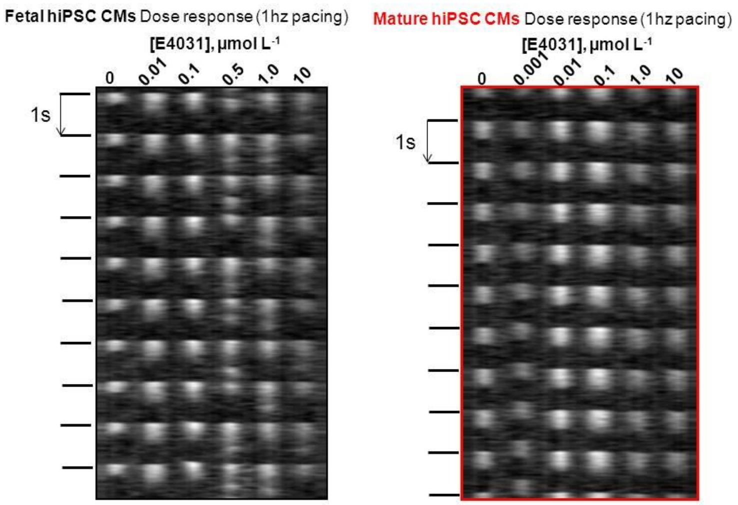

B, Dose Response

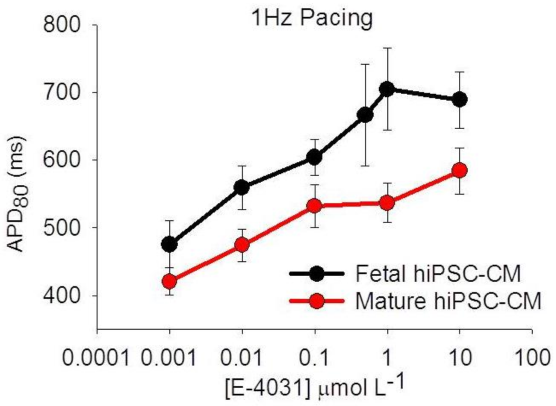

Supplemental figure 7. E-4031 effects in electrically paced iCell hiPSC-CM monolayers. A, Time space plots of a single row across the full range of E-4031 concentrations tested. **B,** Dose response shows that the fetal hiPSC-CM monolayers (black) have longer duration APD_{80} compared to mature (red) monolayers when pacing frequency is matched (1Hz).

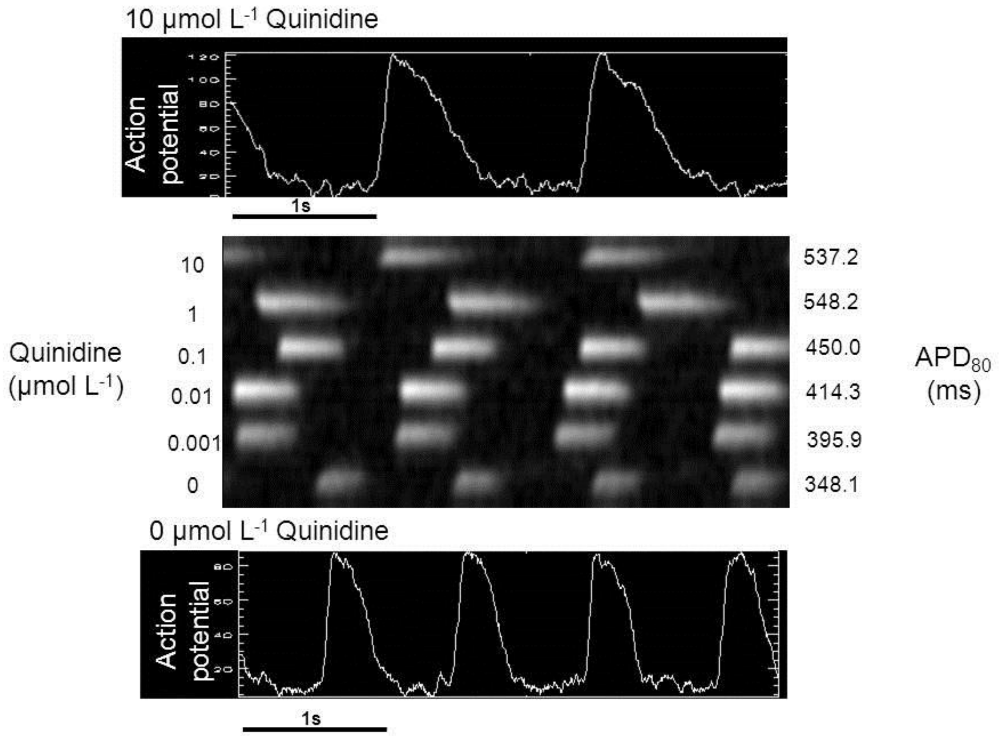

Supplemental figure 8. Validation of platform using quinidine. In mature hiPSC-CM monolayers quinidine (a high risk compound) caused prolongation of the APD in a dose dependent way-as expected. Time 0 is to the left and the single pixel recordings are time matched with the time space plots.

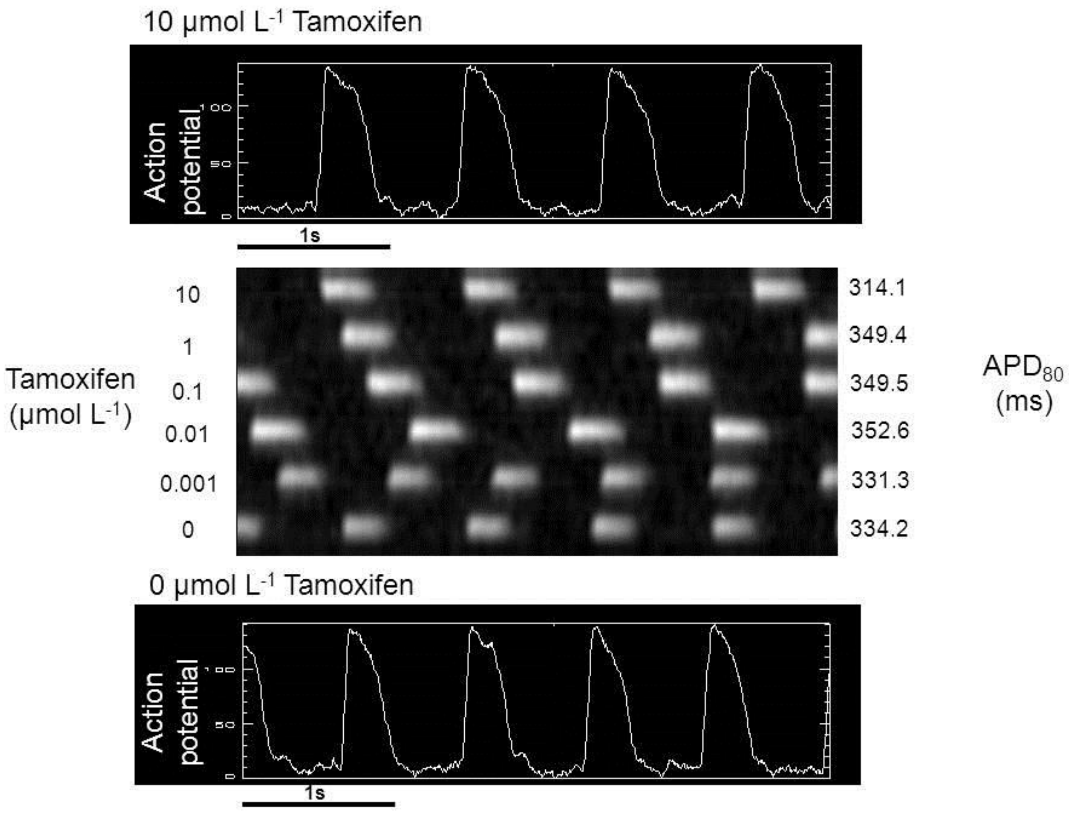

Supplemental figure 9. Validation of platform using tamoxifen. In mature hiPSC-CM monolayers tamoxifen (a low risk compound) did not prolong the APD at any concentration used. Time space plot shows spontaneous action potentials.

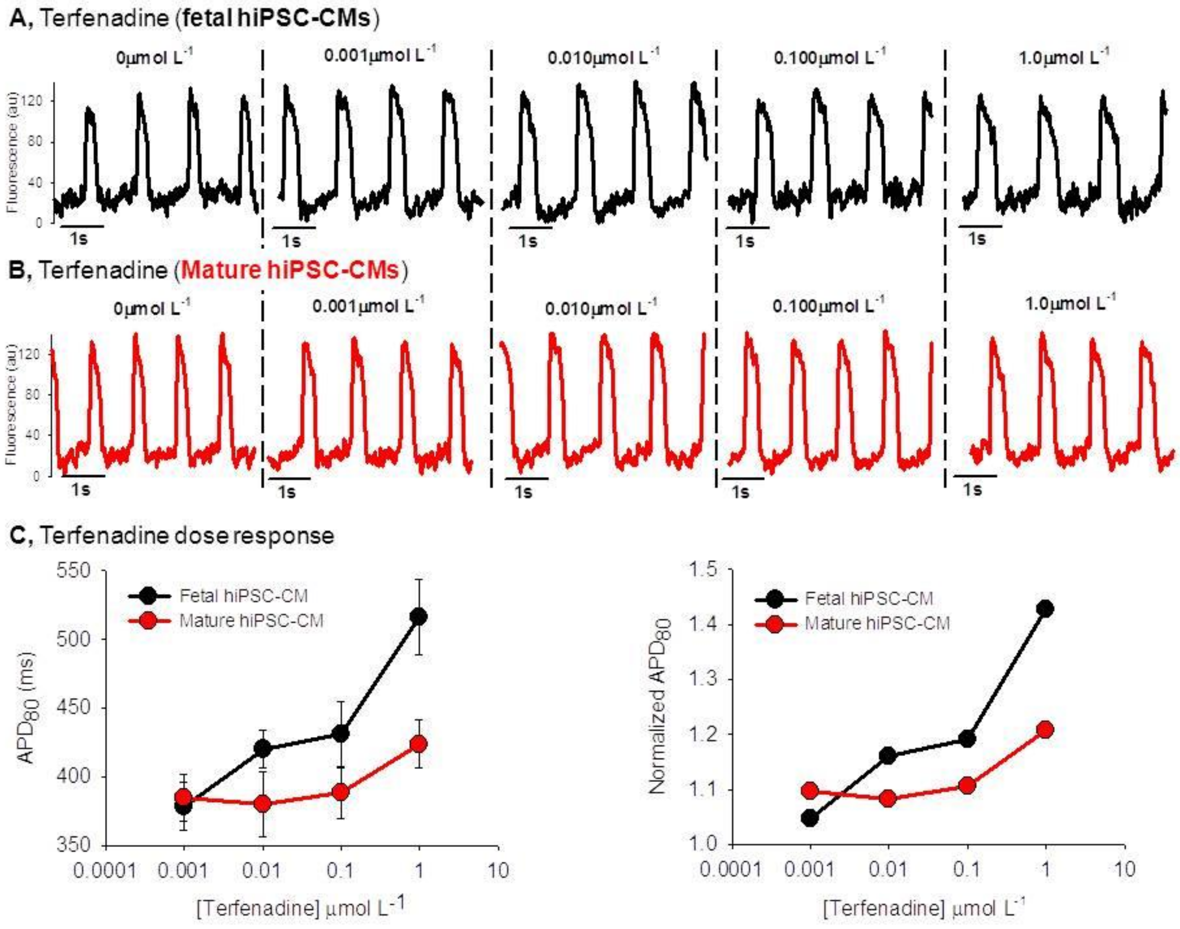

Supplemental figure 10. Dose response for terfenadine. A&B are original recordings of spontaneous action potentials. **C,** Dose response for fetal (black) and mature (red) hiPSC-CM monolayers.
